# Prognostic value of serum C-reactive protein in idiopathic multicentric Castleman disease and construction of a prognostic model for patients

**DOI:** 10.3389/fmed.2025.1544250

**Published:** 2025-04-16

**Authors:** Zhixiang Lei, Ya Wang, Tiantian Yu, Yiting Zhang, Wenting Cui, Cancan Luo, Qingqing Luo, Lili Zhou, Yuchen Gao, Li Yu

**Affiliations:** ^1^The Second Affiliated Hospital, Jiangxi Medical College, Nanchang University, Nanchang, China; ^2^Jiangxi Provincial Children's Hospital, Nanchang, China; ^3^The First People’s Hospital of Jiujiang, Jiujiang, China

**Keywords:** iMCD, risk factors, CRP, nomogram, prognostic assessment

## Abstract

**Background:**

Idiopathic Multicentric Castleman disease (iMCD) is a type of the rare lymphoproliferative diseases. C-reactive protein (CRP) is a well-recognized biomarker of inflammation, frequently exhibits elevated levels in individuals diagnosed with iMCD. However, its prognostic value of this factor in iMCD remains uncertain.

**Methods:**

The clinical manifestations, biochemical information, treatment plan and overall survival time (OS) of 68 iMCD patients with basic information such as age, sex, time of first diagnosis, blood routine and serum CRP level data from 6 medical institutionsin China and abroad were retrospectively analyzed. The median follow-up time of the study was 44.47 months. The serum CRP level was divided into two groups according to the prognostic relationship by X-tile software, and then it was included in the risk model CRP-A for predicting death, together with the age of first visit > 60 years old, Hemoglobin (HGB) ≤ 80g/L, hepatomegaly and/or splenomegaly and plasma cell (PC) type. The predictive ability of the clinical model was evaluated by drawing calibration curve and ROC curve. The factors affecting the level of serum CRP were analyzed.

**Results:**

Using the Kaplan-Meier method, our analysis suggested that a higher serum CRP level (>26.8 mg/L) was associated with worse overall survival in patients (*p* = 0.004). We developed a multivariable prognostic model based on serum CRP levels to assess survival outcomes in iMCD. The discriminative performance of the model for mortality events was validated through calibration plots and receiver operating characteristic (ROC) curves highlighting CRP as a key biomarker associated with disease prognosis. Additionally, analyzing by chi-square test and Fisher’s exact test showed that age, B-symptoms, hypoalbuminemia, ECOG and plasma cell type were significantly associated with high serum CRP level in patients with iMCD, and that fibrinogen levels was positively correlated with CRP level.

**Conclusion:**

High serum CRP levels are associated with a variety of clinical manifestations and laboratory abnormalities.

## Introduction

Castleman disease (CD) is a heterogeneous lymphoproliferative disorder classified histologically into hyaline vascular (HV), plasma cell (PC), and mixed cellularity (MCD) subtypes, with unicentric (UCD) and multicentric (MCD) forms distinguished by lesion distribution (1–3). UCD generally has a favorable prognosis (5-year survival rate > 90%) after local therapy, while MCD often presents with systemic symptoms (fever, weight loss) and is frequently associated with HHV-8 infection, particularly in immunocompromised patients (e.g., HIV-positive individuals) (4–6). Approximately 30–50% of MCD cases are idiopathic multicentric Castleman disease (iMCD), which can range from mild inflammation to life-threatening cytokine storms or organ failure. iMCD is further subdivided into TAFRO syndrome (thrombocytopenia, anemia, myelofibrosis, organomegaly, and renal failure) and non-specific (iMCD-NOS) subtypes, with a 5-year survival rate of 51–77% (7–9).

Its pathogenesis remains unclear but may involve immune dysregulation, viral infections, or paraneoplastic mechanisms (10). Current treatment for iMCD lacks standardized guidelines and relies on risk-stratified strategies proposed by the CDCN (11). Therapeutic options include corticosteroids, anti-CD20/IL-6/IL-6R agents, and immunosuppressants, applied variably based on disease severity (12, 13). Prognostic modeling remains challenging due to disease heterogeneity and limited biomarkers.

Serum C-reactive protein (CRP), a marker of acute inflammation, has emerged as a potential prognostic indicator in iMCD. Previous studies reported elevated CRP levels in MCD patients and its inclusion as a minor criterion in the 2020 CDCN diagnostic consensus (>10 mg/L) (14–16). However, its prognostic significance in iMCD requires validation. This multicenter analysis of 68 patients evaluated CRP’s role in stratifying overall survival and developed a simplified clinical prognostic model for iMCD characterizing CRP-associated survival risk.

## Materials and methods

### Patients and methods

We identified a total of 68 patients who were diagnosed with iMCD between January 2005 and February 2017 at six prominent medical centers, namely the Affiliated Cancer Hospital of Sun Yat-sen University, The First Affiliated Hospital of Zhejiang University, The Second Affiliated Hospital of Zhejiang University, Shandong Cancer Hospital, The University of Texas M.D. Anderson Cancer Center, and Shanxi Cancer Hospital. The diagnosis of iMCD was made based on accepted guidelines that consider histopathologic features observed in biopsy specimens from affected lymph nodes, tissues, or organs. These diagnoses underwent thorough review by at least two experienced pathologists. Patients with co-morbid malignancies or those who tested positive for EBV or HIV were excluded from the study. Additionally, patients with HHV-8-associated diseases such as Kaposi’s sarcoma or primary effusion lymphoma (PEL), POEMS syndrome and those lacking sufficient clinical information were also excluded. This research received approval from our institutional review board and was conducted in accordance with the principles outlined in the Declaration of Helsinki. Patient data, including gender, age, B symptoms, large masses (Defined as a mass greater than 5 cm in diameter), past history, pathology type, laboratory test results, and imaging findings, were recorded and analyzed. Treatment options and overall survival time (OS) were also evaluated. Hepatosplenomegaly, large masses, and edema were confirmed by ultrasound type B (USG-B) or computed tomography (CT) of the involved areas and of superficial lymph nodes or organs; some patients underwent systemic PET-CT. B symptoms were defined as night sweats, a temperature of more than 38°C or weight loss of more than 10% within the past 6 months. The treatment options for iMCD vary depending on the severity of clinical symptoms and drug accessibility. They include combinations of rituximab ± steroid therapy, two or more chemotherapeutic agents, individual chemotherapeutic agents, IL-6 targeted therapy, glucocorticoids alone, immunotherapy, watchful waiting (including surgery).

The follow-up for all patients with iMCD was conducted from the date of initial diagnosis until December 31, 2018, or the time of death or last follow-up. The median duration of follow-up was 44.47 months (95% CI: 29.29 ~ 59.65 months). OS was defined as the time from death, loss to follow-up, or last recorded contact. Follow-up data were primarily obtained from medical records, telephone records, and WeChat records.

### Statistical analysis

Statistical analysis of the data was conducted using IBM SPSS version 25 and R language version 4.0 software. Measurement data following a normal distribution were reported by the mean ± standard deviation, while data not following a normal distribution were expressed using quartiles. The non-parametric rank sum test was employed for analyzing differences between two groups. CRP levels were stratified using X-TILE (Yale University, USA). Univariate survival analysis was performed using the Kaplan–Meier method, and the log-rank test was used to compare survival between the two groups. Survival-related factors were analyzed using univariable and multivariable Cox regression models. The prognostic utility of the model for death in iMCD patients was assessed through a calibration curve analysis the discriminative performance of the model in patient survival at 1-, 3-, and 5-year was evaluated using receiver operating characteristic (ROC) curves. Correlation analyses were conducted using the chi-square test, Fisher’s exact probability method, and Spearman’s rank correlation coefficient. Statistical significance was defined as *p*-values <0.05 in two-sided tests.

### Clinical information and prognostic information

We conducted data analysis on 68 patients with iMCD from 6 medical institutions. Among these patients, 33 (48.53%) were male and 35 (51.47%) were female. The average age of the patients was 45.49 years, and 16.18% (11/68) of them were over the age of 60. In 68 patients with iMCD, 55.88% (38/68) had elevated serum CRP (normal value 0-10 mg/L), and the median CRP was 17.76 mg/L. The median hemoglobin (HGB) was 122 g/L, 16.18% of patients ≤80 g/L, 83.82% of patients >80 g/L. Serum albumen (ALB) is ≤35.00 g/L in 32.43% of patients (12/37). Detailed laboratory results are shown in Table 1.

**Table 1:** The demographic and laboratory characteristics of all iMCD patients.

Clinical factor	Total	*N* (%) or Mean (±SD)
Gender	68	
Male		33 (48.53)
Female		35 (51.47)
Age (years)	68	
>60		11 (16.18)
≤60		57 (83.82)
Pathology	68	
HV		23 (33.82)
PC		43 (63.24)
MIX		2 (2.94)
B symptom	64	
Yes		26 (40.63)
No		38 (59.37)
ECOG score	49	
0		13 (26.53)
1		12 (24.49)
2		13 (26.53)
3		9 (18.37)
4		2 (4.08)
Hepatomegaly and/or splenomegaly	68	
Yes		12 (17.65)
No		56 (82.35)
Hemoglobin (g/L)	68	
≤80		11 (16.18)
>80		57 (83.82)
Large mass	55	
Yes		8 (14.54)
No		47 (85.45)
WBC, Mean(±SD) (×10^9^ /L)		7.07 ± 2.86
HGB, Mean (±SD) (g/L)		116.89 ± 28.11
PLT, Mean (±SD) (×10^9^ /L)		235.42 ± 130.24
LDH, IQR(U/L)		(136.60, 232.55)
CRP, IQR (mg/L)		(3.66, 78.75)
β2-MG, IQR (mg/L)		(1.35, 4.32)
ESR, IQR (mm/H)		(2.70, 47.00)
ALB, IQR (g/L)		(34.00, 43.00)
AKP, IQR (U/L)		(65.50, 102.00)
IgA, IQR (g/L)		(0, 3.70)
IgG, IQR (g/L)		(0, 17.95)
IgM, IQR (g/L)		(0, 1.17)
Fibrinogen, IQR (g/L)		(2.58, 4.78)

HV, hyaline-vascular; PC, plasma cell; MIX, mixed cellular variant; WBC, white blood cell count; HGB, Hemoglobin PLT, platelets; LDH, lactate dehydrogenase; CRP:C-reactive protein; β2-MG, β2-microglobulin; ESR, erythrocyte sedimentation rate; ALB, albumin; AKP, Alkaline phosphatase; IQR, Interquartile range.

By the end of follow-up, the mortality rate of iMCD patients was 29.41% (20/68), the survival rate was 70.59% (48/68), and the survival rate was 63.33% (19/30) in the chemotherapy group (including single-drug chemotherapy, combination chemotherapy, chemotherapy plus surgery). The prognostic information of different treatment regimens is shown in Table 2.

**Table 2:** Treatment and prognosis information for patients with iMCD.

Treatment program	*N*	Evaluation of efficacy				Prognostic information	
		Cases with efficacy assessment	CR / PR (N)	SD (N)	PD (N)	Survival (N)	Death (N)
Chemotherapy group	30	23	14	8	1	19	11
CHOP	17	15	9	5	1	10	7
COP	5	4	1	3	/	3	2
R-CHOP/R-CHOP	4	3	3	/	/	3	1
FC	1	1	1	/	/	1	0
CTX	1	0	/	/	/	1	0
Chemotherapy + surge-ries	2	0	/	/	/	1	1
Steroids	5	4	1	3	/	4	1
R + steroids	1	1	/	/	/	1	0
Anti-IL-6 targeted therapy	2	1	1	/	/	2	0
Stuximab	1	1	1	/	/	1	0
Tolizumab	1	0	/	/	/	1	0
Watchful waiting (with surgery)	30	12	4	5	3	23	7

CHOP, Cyclophosphamide, doxorubicin, vincristine and prednisone; COP, Cyclophosphamide, vincristine and prednisone; R-CHOP:CHOP+ rituximab; FC, Fludarabine, cyclophosphamide; CTX, Cyclophosphamide; CR, Complete remission; PR, Partial remission; SD, Stable disease; PD, Progressive disease.

### Establish a new prognostic model CRP-A for iMCD patients

**Figure 1:**
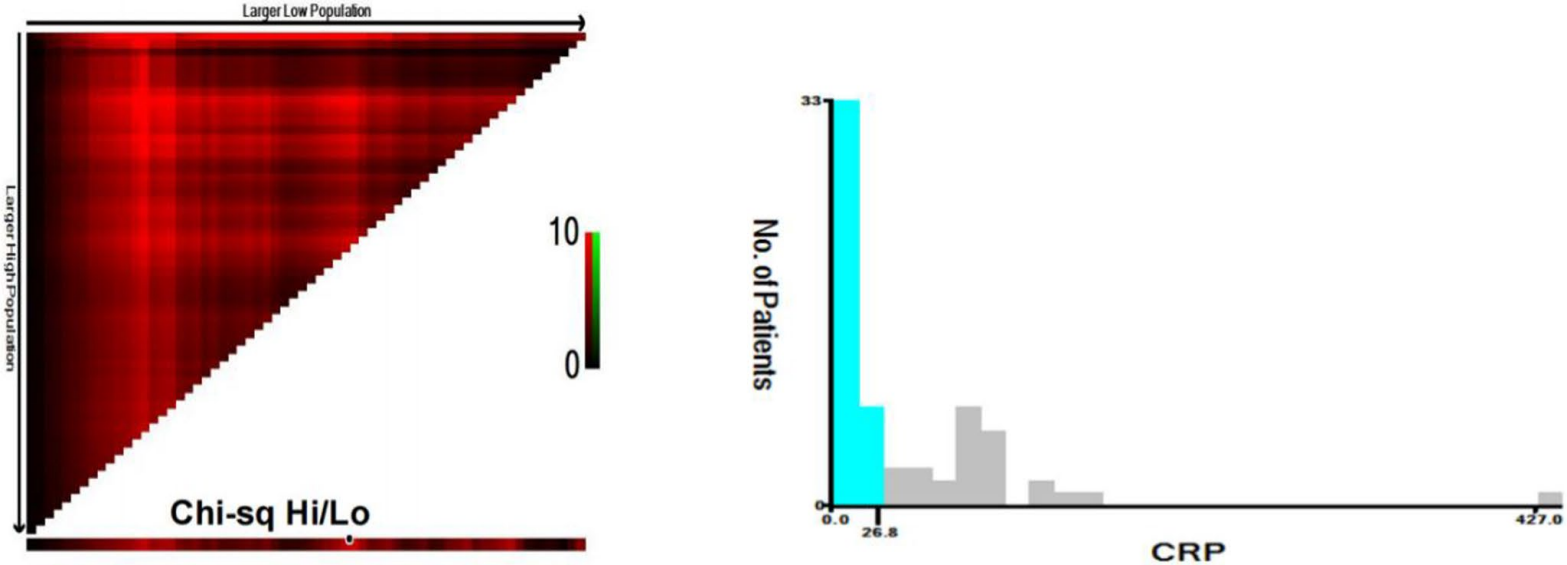
CRP at the best prognostic cutoff value for OS in patients with iMCD.

**Figure 2:**
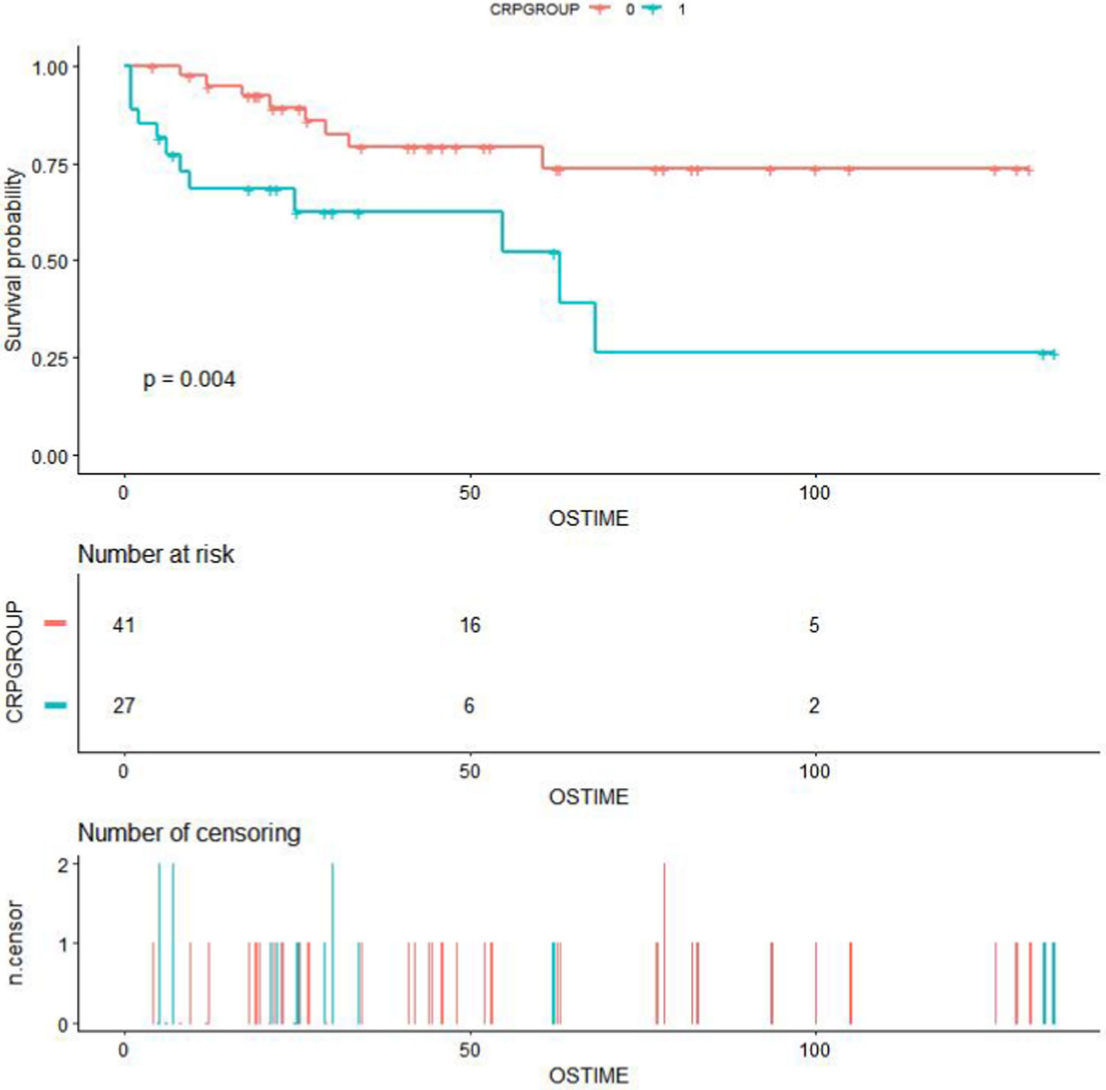
Survival curves for iMCD patients in different CRP groups.

We utilized X-TILE software to stratify serum CRP levels for assessing the outcomes of iMCD patients. The optimal cut-off value for stratification was determined as 26.8 mg/L (Figure 1). A total of 68 iMCD patients were included in this study, among whom 41 had a serum CRP level of ≤26.8 mg/L and 27 had a serum CRP level of >26.8 mg/L. Subsequently, we employed the Kaplan–Meier method to plot survival curves for different serum CRP groups. The results demonstrated that higher levels of serum CRP (>26.8 mg/L) were significantly associated with worse OS in these 68 iMCD patients, with a *p*-value of 0.004 (Figure 2).

**Figure 3:**
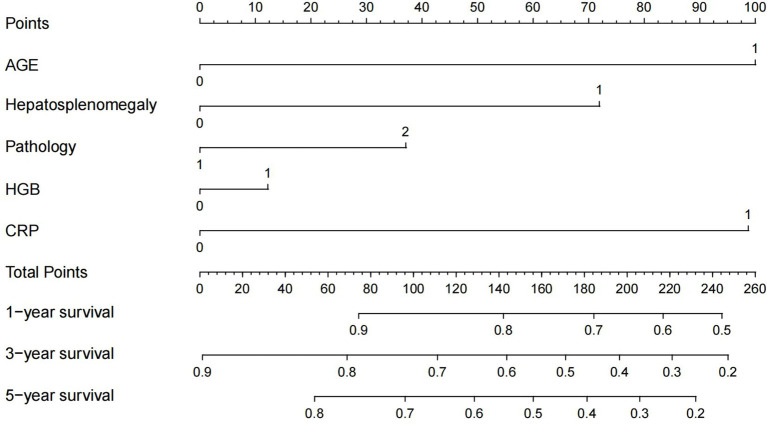
CRP-A nomogram model. Age > 60 years was 1 and ≤ 60 years was 0; presence of hepatomegaly and/or splenomegaly was 1 and absence of hepatosplenomegaly was 0. Pathologic type PC was 2, otherwise 1; HGB(Hemoglobin) ≤ 80 g/L was 1 and HGB >80 g/L was 0; CRP (C-reactive protein) ≤ 26.8 mg/L was 0 and CRP >26.8 mg/L was 1.

As shown in Table 3, the Cox proportional hazards model analysis demonstrated that both age (*p* = 0.004) and CRP levels (*p* = 0.007) exhibited significant associations with OS in univariate analysis. After adjusting for potential confounders including age, tumor histology, hepatosplenomegaly, and serum albumin levels through multivariable Cox regression modeling, elevated CRP levels remained independently associated with shortened overall survival (hazard ratio HR = 2.977, 95% confidence interval CI: 1.139–7.784, *p* = 0.026). These findings suggest that CRP may serve as an independent prognostic biomarker for this patient population. Based on our previously identified prognostic risk factors for iMCD—namely hepatosplenomegaly, HGB ≤80 g/L, age > 60 years, and pathological subtype PC-type—this study innovatively developed the CRP-A prognostic model by integrating CRP measurement (Figure 3). Through systematic incorporation of these four established risk factors alongside CRP variables, the model demonstrated significantly improved the model’s prognostic stratification capacity for survival outcomes in patients with iMCD. In this model, these four elements all receive corresponding scores displayed at the top section of the plot. The total score obtained by summing the scores of all variables corresponds to the score at the bottom of the nomogram. The total risk score derived from the model is aligned with the probability scales to estimate survival probabilities at 1-, 3-, and 5-year post-diagnosis. Within this prognostic stratification framework, age > 60 years and CRP levels >26.8 mg/L contribute the highest weights to the composite risk score, followed by hepatosplenomegaly and PC-type pathology, while HGB <80 g/L demonstrates a comparatively lower impact.

**Table 3:** Univariate and multivariate Cox hazards analysis for OS in iMCD.

		Univariate analysis			Multivariate analysis		
Clinical features		HR	95% CI	*p*	HR	95% CI	*p*
Age, y	>60VS ≤ 60	4.358	(1.612–11.783)	0.004*	2.998	(1.007–8.345)	0.036*
Hepatosplenomegaly	Yes VS No	1.738	(0.667–4.531)	0.258	2.236	(0.082–6.234)	0.124
Pathology	Yes VS No	1.968	(0.812–4.771)	0.134	1.522	(0.582–3.98)	0.392
HGB(g/L)	>80VS ≤ 80	1.692	(0.563–5.085)	0.349	1.124	(0.349–3.618)	0.844
CRP(mg/L)	>26.8VS ≤ 26.8	3.463	(1.406–8.53)	0.007*	2.977	(1.139–7.784)	0.026*

HGB, Hemoglobin; CRP, C-reactive protein; ALB, albumin.

*p-value of < 0.05 was considered statistically significant.

**Figure 4:**
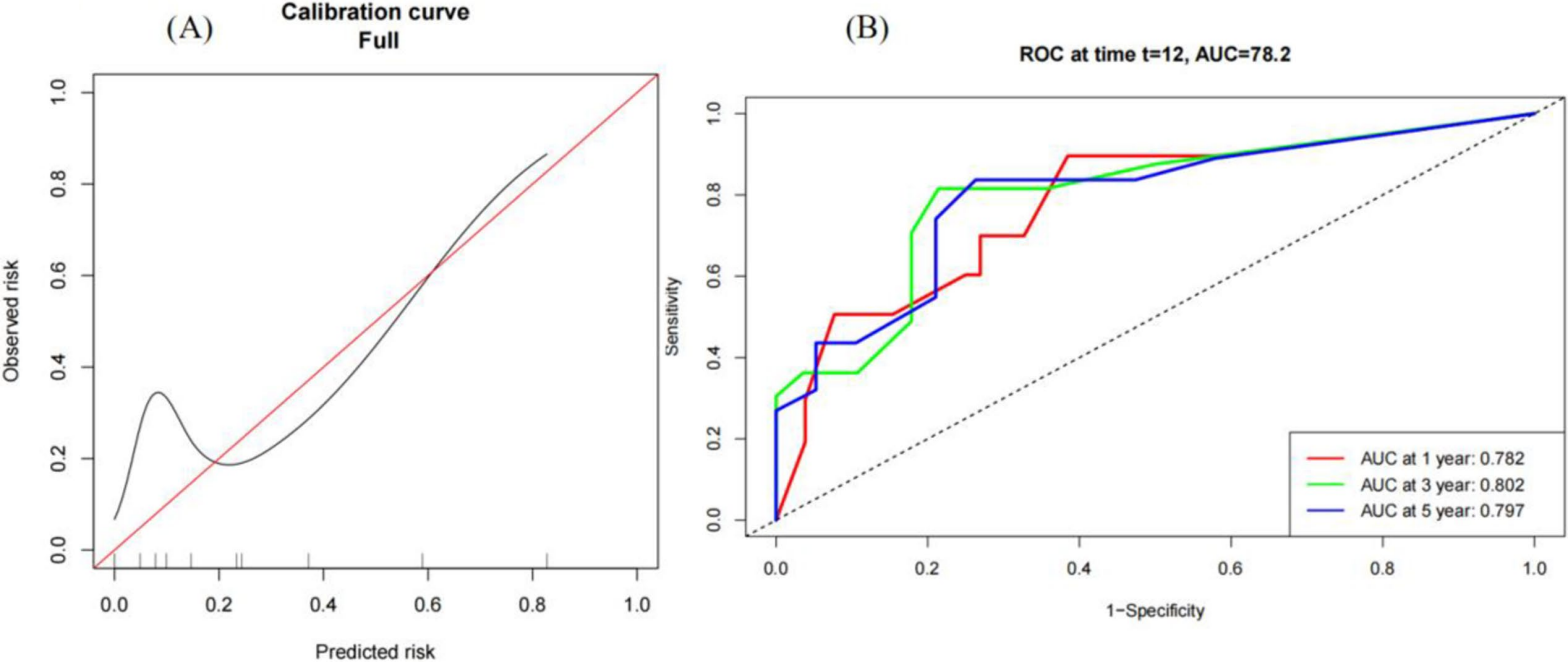
Calibration curve of the CRP-A model for the predicted risk of death in patients with iMCD. **(A)** Calibration curve of the predicted risk of death and actual risk of death in iMCD patients by CRP-A model. **(B)** ROC curves for CRP-A model predicting survival at years 1, 3 and 5 in patients with iMCD.

To evaluate the effectiveness of the modeling results, we created calibration plots of CRP-A model stratifying death in patients with iMCD at year 1, 3, and 5 after diagnosis. The calibration plots indicated close alignment between model-derived and observed outcomes, while ROC curves demonstrated high discriminative accuracy, especially when the model-derived risk of death was ≥0.2, the model had a high the model showed high discriminative accuracy for mortality events for the actual death rate (Figure 4A). The ROC curve was then plotted, revealing that the prognostic model CRP-A prognostic model exhibited favorable discriminative accuracy for survival. The corresponding area under the curve (AUC) values for the 1st, 3rd, and 5th were determined to be 0.782, 0.802 and 0.797, respectively (Figure 4B).

### Correlation analysis of serum CRP

We employed the chi-square test and the Fisher exact probability method to examine the associations between different serum CRP groups (the threshold value was set at 26.8 mg/L) and age groups (the threshold age is set at 60 years), serum ALB groups (The cut-off value was 35 g/L), HGB groups (The cut-off value was 80 g/L), as well as the relationship between serum CRP and ECOG ≥2, liver and/or splenomegaly, large masses, and PC type. The results showed that age > 60 years (*p* = 0.035), presence of B symptoms (*p* = 0.002), hypoalbuminemia (*p* = 0.008), ECOG ≥2 (*p* < 0.001) and pathological type PC (*p* = 0.008) were associated with high serum CRP level (CRP >26.8 mg/L). However, the different HGB groups (*p* = 0.151), large masses (*p* = 0.585) and the presence of liver and/or splenomegaly (*p* = 0.863) showed no significant association with serum CRP levels (Table 4). In addition, we analyzed the distribution of HGB, age, and albumin among patients with different CRP groups, which showed that patients with high serum CRP levels (CRP >26.8 mg/L) tended to have lower HGB, albumin levels, and a higher age at first diagnosis.

**Table 4:** Correlation analysis of serum CRP levels in patients with iMCD.

Clinical factor	Total	CRP> 26.8 mg/ L *N* (%)	CRP ≤ 26.8 mg/L N(%)	X2	*p*
Age (years)	68				
>60	11	8 (72.73)	3 (27.27)	4.445 ^a^	**0.035**
≤60	57	19 (33.33)	38 (66.67)		
HGB	68				
≤ 80 g/L	11	7 (63.64)	4 (36.36)	2.060 ^a^	0.151
> 80 g/L	57	20 (35.09)	37 (64.91)		
B symptom	64				
Yes	26	16 (61.54)	10 (38.46)	9.293	**0.002**
No	38	9 (23.68)	29 (76.32)		
Hepatomegaly and/or splenomegaly	68				
Yes	12	4 (33.33)	8 (66.67)	0.030 ^a^	0.863
No	56	23 (41.07)	33 (58.93)		
ALB	37				
≤35 g/L	12	6 (50.00)	6 (50.00)		**0.008** ^**b**^
>35 g/L	25	2 (8.00)	23 (92.0)		
ECOG≥2	49				
Yes	24	17 (70.83)	7 (29.17)	12.790	**<0.001**
No	25	5 (20.00)	20 (80.00)		
Large mass	55				
Yes	8	2 (25.00)	6 (75.00)	0.299	0.585
No	47	20 (42.55)	27 (57.45)		
PC type	55 ^c^				
Yes	30	15 (50.00)	15 (50.00)	6.971	**0.008**
No	25	4 (16.00)	21 (84.00)		

a *N > 40, when 1 < T < 5, the four-cell table continuity correction test was selected.*

b *N < 40, data were correlated using Fisher’s exact probability method.*

c Pathology type excludes unclassified.

HGB, Hemoglobin; CRP, C-reactive protein; ALB, albumin; ECOG, Eastern Cooperative Oncology Group.

*p-value of < 0.05 was considered statistically significant.

Spearman rank correlation analysis was used to examine the correlation between continuous biochemical indicators in iMCD patients and the total serum CRP levels in patients. The results revealed a significant negative correlation between HGB levels (*p* < 0.001), serum albumin levels (*p* < 0.001), and serum IgA levels (*p* = 0.023) in iMCD patients with the overall CRP level. Conversely, there was a positive correlation observed between fibrinogen levels (*p* = 0.025) and ECOG scores (*p* = 0.002) and the overall CRP levels.

## Discussion

The identification of prognostic factors and development of prognostic models are imperative for enhancing treatment decisions and facilitating early prognosis. While the research on prognostic factors for iMCD remains relatively limited, further investigation is warranted. As a whole, our study determined the clinical characteristics and prognostic outcomes of 68 patients with CD with different serum CRP levels. Our study found that high serum CRP level (>26.8 mg/L) was a risk factor for the survival of patients with iMCD. we built the CRP-A nomogram model based on serum CRP level (>26.8 mg/L), age > 60 years, hepatomegaly and/or splenomegaly, HGB ≤80 g/L, and PC type, which demonstrated robust discriminative performance in stratifying the 1-year, 3-year, and 5-year survival of iMCD patients. The aim of this study was to identify the correlation between clinical presentation, prognostic factors and serum CRP levels in patients with iMCD, and to construct a simple clinical model for stratifying mortality in patients with iMCD.

Serum CRP, an acute phase protein synthesized by the liver, not only reflects the degree of inflammatory response in the body at an early stage, but also have been shown to be a prognostic indicator for certain tumors in several studies (17, 18). As a minor criterion for the diagnosis of CD and an adverse prognostic factor in patients with CD identified by CDCN, serum CRP has been studied in relatively few cases for prognostic analysis in patients with iMCD. In the present study, we observed a significant association between higher serum CRP levels and inferior OS in patients (*p* = 0.004). The optimal cut-off value of serum CRP was found to be 26.8 mg/L as determined using the X-TILE software. Elevated serum CRP levels represent the presence of inflammatory response in patients, and the tendency to anemia may be due to the fact that most patients with iMCD also have elevated expression of serum IL-6 in inflammatory states, which has been found to be a major inducer of the production of hepcidin, a key regulator of iron homeostasis *in vivo*, and whose increase is associated with inflammatory anemia (19).

The above data indicate that CRP serves as a simple and effective indicator reflecting the disease severity and prognosis of iMCD. We then converted CRP levels into categorical variables based on a threshold of 26.8 mg/L (<26.8 mg/L and ≥ 26.8 mg/L). A nomogram model was developed, incorporating factors such as age, hepatic and/or splenic enlargement, hemoglobin (HGB) levels, and pathology type. Previous studies have shown that age > 60 years and the presence of splenomegaly are prognostic factors for progression-free survival (20). Furthermore, in a previous study conducted by our research team, we identified hepatomegaly and/or splenomegaly, HGB levels ≤80 g/L, age > 60 years, and the presence of PC pathology type as significant prognostic risk factors for patients with iMCD (21). The CRP-A model of this study and the previous iMCD-IPI model are prognosis risk assessment models for iMCD patients, and they have certain clinical guiding value. The difference is that the CRP-A model includes serum CRP, a laboratory indicator that impacts the patient’s OS, making it convenient and straightforward for clinical assessment. Subsequently, we validated the CRP-A prognostic model, both ROC curves and calibration curves showed that the nomogram discriminative accuracy in stratifying the presence of a significant number of patients. ROC curves and calibration curves demonstrated robust discriminative performance for survival outcomes in patients with iMCD. In this model, patients with serum CRP levels >26.8 mg/L and age at diagnosis >60 years were of primary importance and equal importance in stratifying their 1-, 3-, and 5-year mortality risk. This suggests that serum CRP should be routinely measured in patients with iMCD in the future, and that early control of the inflammatory response in patients with markedly elevated CRP may improve their long-term survival. Therefore, implementing early intervention strategies may enhance long-term survival in these patients.

We further analyzed the correlation of serum CRP levels with other specific clinical characteristic in iMCD patients. Our results showed that iMCD patients with elevated serum CRP levels were more likely to have B symptoms and higher ECOG scores than those with low CRP levels, which is consistent with the fact that patients with iMCD usually have a systemic inflammatory response. Serum ALB levels, HGB levels, and IgA levels were negatively correlated with serum CRP. Some earlier studies have verified that STAT3 is a major transcription factor that upregulates ferritin during inflammation via the IL-6 transduction pathway (22). Antibodies against the IL-6 receptor significantly improved systemic manifestations of MCD, including HGB levels, by blocking serum IL-6 (23). High levels of serum CRP are associated with hypoalbuminemia, which may be due to decreased hepatic capacity to synthesize albumin as a result of increased expression of a range of inflammatory factors. Elderly (>60 years of age at first diagnosis) iMCD patients are more likely to have high CRP levels than younger (≤60 years of age at first diagnosis) patients, and therefore controlling the inflammatory response in elderly patients is key to treatment. iMCD patients with PC-type desease are more likely to have elevated CRP, perhaps related to humoral immunity involving plasma cell infiltration in the lymph node growth centers of patients with PC-type iMCD.

Several limitations are associated with our study. First, this is a retrospective study with some missing data. Furthermore, some patients without obvious symptoms or with mild symptoms chose to wait for treatment under observation, while CHOP and R-CHOP were common among chemotherapy regimens. The number of patients treated with single-agent chemotherapy, steroidal single-agent therapy, and single-agent therapy with rituximab was relatively small; therefore, no comparative analysis was conducted to evaluate the OS of the various treatment regimens; In addition, all cases of treatment with IL-6 targeted therapy were foreign patients, and none of the included domestic patients were treated with IL-6 targeted therapy; Due to the rarity of this disease, the value of the prognostic model has not been validated by external cohorts, which poses certain limitations. The repetition of cases treated with IL-6-targeted therapy in both foreign and domestic settings further emphasizes the lack of external validation for the prognostic model, highlighting this limitation.

## Conclusion

Our study determined the clinical characteristics and prognostic outcomes of 68 patients with CD with different serum CRP levels. Our study found that high serum CRP level (>26.8 mg/L) was a risk factor for the survival of patients with iMCD; and we built the CRP-A nomogram model based on serum CRP level (>26.8 mg/L), age > 60 years, hepatomegaly and/or splenomegaly, HGB ≤80 g/L, and PC type, which can better quantify survival risk of the 1-year, 3-year, and 5-year iMCD patients, providing a tool for risk stratification in iMCD based on CRP-associated biomarkers. Our study will clarify our understanding of MCD and that the unfavorable risk factors for serum CRP in this study will inform therapeutic decisions and prognostic assessment. During future studies, it attaches a great importance with exploring their etiology, classification, treatment, prognosis and so on.

## Data Availability

The raw data supporting the conclusions of this article will be made available by the authors, without undue reservation.

## References

[1] Carbone A Borok M Damania B Gloghini A Polizzotto MN Jayanthan RK. Castleman disease. Nat Rev Dis Prim. (2021) 7:1:84. doi: 10.1038/s41572-021-00317-7, PMID: 34824298 PMC9584164

[2] Geuna M Riccardo B Cignetti A Santoro N Gueli A Elia AR. Normal circulating B cells are markedly reduced in follicular lymphoma and diffuse large B cell lymphoma at diagnosis. Blood. (2014) 124:2970–09. doi: 10.1182/blood.V124.21.2970.2970, PMID: 39837500

[3] Wojtyś M Piekarska A Kunc M Ptaszyński K Biernat W Zaucha JM. Clinicopathological comparison and therapeutic approach to Castleman disease—a case-based review. J Thorac Dis. (2019) 11:4859–74. doi: 10.21037/jtd.2019.10.73 31903277 PMC6940266

[4] Dispenzieri A Armitage JO Loe MJ Geyer SM Allred J Camoriano JK. The clinical spectrum of Castleman's disease. Am J Hematol. (2012) 87:997–1002. doi: 10.1002/ajh.23291, PMID: 22791417 PMC3900496

[5] Fajgenbaum DC Uldrick TS Bagg A Frank D Wu D Srkalovic G. International, evidence-based consensus diagnostic criteria for HHV-8–negative/idiopathic multicentric Castleman disease. Blood. (2017) 129:1646–57. doi: 10.1182/blood-2016-10-746933, PMID: 28087540 PMC5364342

[6] Barozzi P Luppi M Cagossi K Maiorana A Marasca R Artusi T. The oncogenic 30 and 69 bp deletion variants of the EBV LMP-1 gene are common in HIV-negative lymphoproliferations, both malignant and benign. Ann Oncol. (1999) 10:467–9. doi: 10.1023/A:1008381006612, PMID: 10370791

[7] Yu L Tu M Cortes J Xu-Monette ZY Miranda RN Zhang J. Clinical and pathological characteristics of HIV-and HHV-8–negative Castleman disease. Blood. (2017) 129:1658–68. doi: 10.1182/blood-2016-11-748855, PMID: 28100459 PMC5364343

[8] Zhang X Rao H Xu X Li Z Liao B Wu H. Clinical characteristics and outcomes of Castleman disease: a multicenter study of 185 Chinese patients. Cancer Sci. (2018) 109:199–206. doi: 10.1111/cas.13439, PMID: 29124835 PMC5765290

[9] Phillips AD Kakkis JJ Tsao PY Pierson SK Fajgenbaum DC. Increased mTORC2 pathway activation in lymph nodes of iMCD-TAFRO. J Cell Mol Med. (2022) 26:3147–52. doi: 10.1111/jcmm.17251, PMID: 35488725 PMC9170805

[10] Goodman AM Jeong AR Phillips A Wang HY Sokol ES Cohen PR. Novel somatic alterations in unicentric and idiopathic multicentric Castleman disease. Eur J Haematol. (2021) 107:642–9. doi: 10.1111/ejh.13702, PMID: 34431136 PMC9624446

[11] van Rhee F Voorhees P Dispenzieri A Fosså A Srkalovic G Ide M. International, evidence-based consensus treatment guidelines for idiopathic multicentric Castleman disease. Blood. (2018) 132:2115–24. doi: 10.1182/blood-2018-07-862334, PMID: 30181172 PMC6238190

[12] Abramson JS. Diagnosis and Management of Castleman Disease. J Natl Compr Cancer Netw. (2019) 17:1417–9. doi: 10.6004/jnccn.2019.5037, PMID: 31766018

[13] Iwaki N Fajgenbaum DC Nabel CS Gion Y Kondo E Kawano M. Clinicopathologic analysis of TAFRO syndrome demonstrates a distinct subtype of HHV-8-negative multicentric Castleman disease. Am J Hematol. (2016) 91:220–6. doi: 10.1002/ajh.24242, PMID: 26805758

[14] Hu C Zou Y Pan J Yang J Yang T Tan T. Analysis of clinical characteristics, pathological changes and changes of Interleukin-6 (IL-6) and C-reactive protein (CRP) in children with Castleman's disease. Med Sci Monit. (2020) 26:e924783. doi: 10.12659/MSM.924783 32873770 PMC7446284

[15] Shiroshita K Kikuchi T Okayama M Kasahara H Kamiya T Shimizu T. Interleukin-6-producing intravascular large B-cell lymphoma with lymphadenopathy mimicking the histology of multicentric Castleman disease. Intern Med. (2020) 59:3061–5. doi: 10.2169/internalmedicine.5046-20, PMID: 32759587 PMC7759707

[16] Dispenzieri A Fajgenbaum DC. Overview of Castleman disease. Blood. (2020) 135:1353–64. doi: 10.1182/blood.2019000931, PMID: 32106302 PMC13174927

[17] Zhang Y Gu D. Prognostic impact of serum CRP level in head and neck squamous cell carcinoma. Front Oncol. (2022) 12:889844. doi: 10.3389/fonc.2022.889844, PMID: 35847918 PMC9277075

[18] Minichsdorfer C Gleiss A Aretin MB Schmidinger M Fuereder T. Serum parameters as prognostic biomarkers in a real world cancer patient population treated with anti PD-1/PD-L1 therapy. Ann Med. (2022) 54:1339–49. doi: 10.1080/07853890.2022.2070660, PMID: 35535695 PMC9103267

[19] Verga Falzacappa MV Vujic Spasic M Kessler R Stolte J Hentze MW Muckenthaler MU. STAT3 mediates hepatic hepcidin expression and its inflammatory stimulation. Blood. (2007) 109:353–8. doi: 10.1182/blood-2006-07-033969, PMID: 16946298

[20] Dong Y Wang M Nong L Wang L Cen X Liu W. Clinical and laboratory characterization of 114 cases of Castleman disease patients from a single Centre: paraneoplastic pemphigus is an unfavourable prognostic factor. Br J Haematol. (2015) 169:834–42. doi: 10.1111/bjh.13378, PMID: 25824806

[21] Yu L Shi M Cai Q Strati P Hagemeister F Zhai Q. A novel predictive model for idiopathic multicentric Castleman disease: the international Castleman disease consortium study. Oncologist. (2020) 25:963–73. doi: 10.1634/theoncologist.2019-0986, PMID: 32852137 PMC7648372

[22] Pietrangelo A Dierssen U Valli L Garuti C Rump A Corradini E. STAT3 is required for IL-6-gp130-dependent activation of hepcidin in vivo. Gastroenterology. (2007) 132:294–300. doi: 10.1053/j.gastro.2006.10.018, PMID: 17241879

[23] Nishimoto N Kanakura Y Aozasa K Johkoh T Nakamura M Nakano S. Humanized anti-interleukin-6 receptor antibody treatment of multicentric Castleman disease. Blood. (2005) 106:2627–32. doi: 10.1182/blood-2004-12-4602, PMID: 15998837

